# Impact of Birth Companionship on Maternal and Fetal Outcomes in Primigravida Women in a Government Tertiary Care Center

**DOI:** 10.7759/cureus.38497

**Published:** 2023-05-03

**Authors:** Kanak Dubey, Neeraj Sharma, Dolly Chawla, Ritu Khatuja, Sandhya Jain

**Affiliations:** 1 Obstetrics and Gynecology, Dr. Babasaheb Ambedkar Medical College and Hospital, New Delhi, IND; 2 Obstetrics and Gynecology, All India Institute of Medical Sciences, Rishikesh, Rishikesh, IND

**Keywords:** positive childbirth experience, labour companionship, spontaneous vaginal birth, maternal satisfaction, childbirth experience

## Abstract

Background: Studies have shown that the support provided by birth companions during labour and childbirth improves maternal and neonatal outcomes and helps women in having a positive childbirth experience.

Objective: To study the role of birth companionship on maternal and foetal outcomes along with their satisfaction rates in primigravidas supported by birth companions compared to those receiving routine care in the labour room.

Design: The study was a prospective comparative observational study. One hundred fifty primigravida women were randomly divided into two groups, one group (n = 75) who were allowed a female companion of their choice (A) and the other group (n = 75) who did not have a birth companion receiving routine standard care as routinely practised in our hospital. The data was entered in MS Excel Sheet and analysis was done using the statistical package for social sciences (SPSS) version 21.0 (IBM Corp., Armonk, NY). A p-value of <0.05 was considered statistically significant.

Results: Spontaneous vaginal births were significantly higher in group A (Group A: mean = 67; SD = 89.3%, Group B: mean = 57; SD = 76 %; p = 0.03) along with a favourable trend in a shorter duration of the first stage of labour (9.96±1.09 vs 11.95±3.11 hours) and decreased need of augmentation (Group A; mean = 10; SD = 13.3, Group B: mean = 23; SD = 30.7; p = 0.01). Maternal satisfaction was significantly higher in group A (Group A: mean = 60; SD = 80, Group B: mean = 44; SD = 58.7; p <0.01) and in the early initiation of breastfeeding (Group A: mean = 70; SD = 93.3, Group B: mean = 55; SD = 73.3; p <0.01). No statistical difference was observed between the mean duration of labour, mode of delivery, and NICU stay of the baby.

Conclusions: Companionship during labour may improve outcomes for women and infants, including increased spontaneous vaginal birth, shorter labour duration, a low five-minute Apgar score, and higher satisfaction rates with childbirth experiences.

## Introduction

Women have the right to be supported and to receive respectful maternity care (RMC) during childbirth, which is undoubtedly one of the most remarkable events in her life [1]. Access to quality services is not guaranteed, especially in developing countries like India; the absence of services, lack of attention during childbirth, and disrespectful treatment may compromise care [2-4]. Women may decline to receive care due to previous disrespectful care; they may also discourage others from receiving care even if the clinician is skilled at handling difficulties [4-6]. A critical component of global efforts to reduce maternal mortality is ensuring that all women have access to skilled RMC before, during, and after childbirth [7].

Staff shortages are common in hospitals in developing and densely populated countries such as ours, resulting in labouring women having minimal contact with midwives because of the poor healthcare worker-population ratio. Birth companionship has been introduced and is currently being advocated for expansion in 2,235 facilities including 179 medical college facilities in India under the “Laqshya Scheme” (Labour room and maternity OT quality improvement initiative) [8].

Positive results for intrapartum, perinatal, and neonatal outcomes have been demonstrated with non-medical labour assistance, also known as Doulas in the US [9,10]. Increased vaginal delivery rates, fewer analgesic needs, shorter labours, higher mother satisfaction and better breastfeeding initiation within the first 24 hours were all clearly beneficial [11,12]. As per guidelines of the Laqshya initiative, awareness programmes and training were organized in our set-up to allow the presence of birth companions with primigravida women in labour.

## Materials and methods

We conducted a prospective comparative observational study in the Department of Obstetrics and Gynaecology, in our institute in New Delhi from 2019 to 2021 over a period of 15 months. One hundred fifty booked primigravida patients between 37 and 42 weeks of pregnancy who met the inclusion/exclusion criteria were included in the study.

Data collection and methods

Following the receipt of a well-explained written consent from the patient, the patients were randomly assigned to one of two groups in latent labour, 75 in each, using a block randomised sequence. Randomization was used to conceal allocations and divide the participants into two groups. Group A (n=75) was the primigravida women who were asked to choose a female birth companion of their own choice, preferably the one who had childbirth experience. Group B (n=75) were not accompanied by a birth companion and received standard care in the labour room from hospital staff which includes clear communication by the staff, treating them with respect and dignity, pain relief strategies, and allowing them to mobilise and choose the comfortable position of their choice. 

The study began during the latent phase of labour and included primigravida women 37 to 42 weeks of gestation, singleton live pregnancy, cephalic presentation, the female companion of her choice, able to understand Hindi, and asymptomatic female family relatives, who were Rapid antigen test negative. During labour admission, a brief 20-minute orientation session for birth companions in Group A was held to explain the instructions that would be taken to provide labour support. To take precautions during COVID-19, these birth companions were provided sterile aprons, shoe covers, caps, and masks. Assistance provided by the companion was discussed under the following points:

1. Emotional support (constant presence, reassurance, and praise), information about signs and symptoms of labour; and the progress of labour.

2. Coping techniques and comfort measures like back massage - softly stroking the back in the first stage of labour in both sitting and lying down positions.

3. Breathing exercises that include co-ordinated breathing to prevent the mother from hyperventilating and encouraging mobility.

4. Considerations in addressing the female; speak softly to the mother and speak on behalf of the woman when necessary.

5. Promoting adequate fluid intake and output; reminding the mother to drink water and to keep the bladder empty.

6. Providing a positive perception to the mother to not regard labour as a painful process but rather as the process of giving birth to another human being.

The primary outcome, in the form of the mode of delivery and duration of the first, second and third stages of labour, was noted and recorded in the proforma. Secondary outcomes were evaluated by noting the APGAR score at five minutes of age, the NICU stay, and satisfaction rates in experiencing labour by asking questions to each patient via a self-designed questionnaire after 12 hours of delivery, as well as the time taken by a birth companion to introduce the breastfeeding, was also noted. In assessing the satisfaction rates, a score of yes to more than 5 questions was considered satisfactory.

Details of the birth companion including name, the relation of the companion with the expectant woman, age of the companion and education status were recorded. All findings were recorded in a pre-designed proforma. The data were analysed and statistical tests were applied.

Data analysis

Categorical variables were presented in number and percentage (%) and continuous variables were presented as mean ± SD and median. The normality of data was tested by the Kolmogorov-Smirnov test. If the normality is rejected, then a nonparametric test was used. Statistical tests were applied as followed:

1. Quantitative variables were compared using the unpaired t-test/Mann-Whitney U test (when the data sets were not normally distributed) between the two groups.

2. Qualitative variables were compared using the Chi-square test/Fisher’s exact test.

A p-value of <0.05 was considered statistically significant. The data were entered in the MS EXCEL spreadsheet and analysis was done using Statistical Package for Social Sciences (SPSS) version 21.0 (IBM Corp., Armonk, NY).

Ethical considerations

This study received ethical approval from a nationally recognized ethical review committee in India.

## Results

A total of 150 primigravida women were enrolled in the study, 75 in each, group A and group B. No significant differences were found between groups concerning demographic and obstetric variables. The mean value of age (in years) of group A was 23.84±4.15 and of group B was 23.83±4.03 (p-value 0.98). All were primigravida and married. The mean value of gestational age (in weeks) of groups A and B was 39.36±1.09 and 39.37±1.08, respectively. A statistically significant difference was seen in the need for augmentation of labour with oxytocin between the two groups (13.3% vs 30.7%; p-value 0.01) (Table 1).

**Table 1:** Need of augmentation of two study groups

Need of augmentation	Group A (n=75)		Group B (n=75)		P-value
	No.	%	No.	%	
No	65	86.7	52	69.3	0.01
Yes	10	13.3	23	30.7	

**Figure 1:**
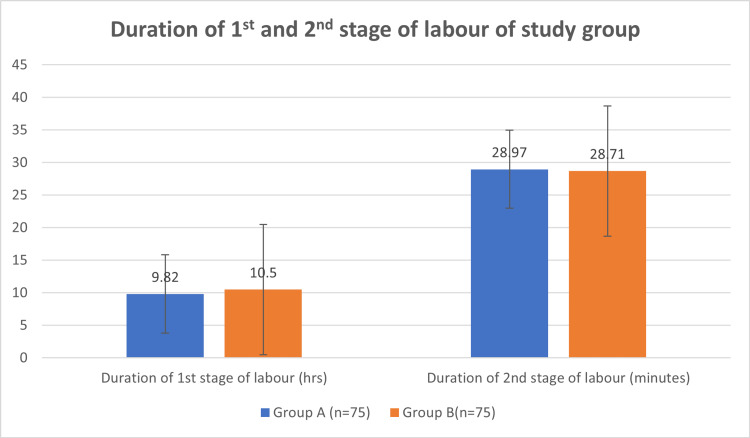
Distribution of mean duration of first and second stages of labour of two study groups

In group B, the duration of labour was 10.5±2.54 hr, as compared to group A (9.82±1.89 hr) in first stage of labour (p-value 0.06). The mean duration of second stage of labour in group A and group B in minutes was 28.97±11.67 and 28.71±11.06, respectively (p-value 0.98) (Table 2, Figure 1).

**Table 2:** Distribution of mean duration of first and second stage of labour of two study groups

	Group A ( Mean ± SD)	Group B (Mean ± SD)	P-value
Duration of 1^st^ stage of labour (hours)	9.82±1.89	10.5±2.54	0.06
Duration of 2^nd^ stage of labour (minutes)	28.97±11.67	28.71±11.06	0.98

The presence of a birth companion was seen to shorten the mean duration of first stage of labour by around 70 minutes, without statistical correlation, calculated using the Mann-Whitney U test. The mean duration of third stage of labour in group A was 10.31±3.03 and group B was 10.31±3.03 in minutes (p-value 0.72) (Table 3).

**Table 3:** The distribution of mean duration of third stage of labour (in minutes) of two study groups

	Group A (n=75)	Group B (n=75)
Duration of 3^rd^ stage of labour (minutes)	10.31	10.48

**Figure 2:**
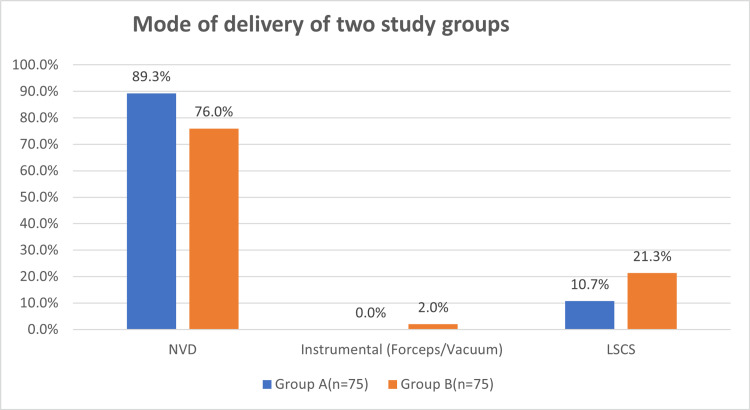
Distribution of mode of delivery between two study groups

No statistical correlation was seen between the duration of the first, second and third stages of labour in both groups in presence of a birth companion. Normal vaginal delivery was higher in group A (89.3%) vs group B (76%), (p-value 0.03) (Table 4, Figure 2).

**Table 4:** Mode of delivery in two study groups

Mode of delivery	Group A (n=75)		Group B (n=75)		P-value
	No.	%	No.	%	
NVD	67	89.3	57	76.0	0.03
Instrumental (Forceps/Vacuum)	0	0.0	2	2.7	0.49
LSCS	8	10.7	16	21.3	0.07

No statistical significance was present between the rates of caesarean sections and instrumental deliveries. Group A had lower caesarean section rates (10.7%) and lower instrumental deliveries when compared with group B (p-value of 0.49 and 0.07) using Chi-square test. The mean Apgar scores at five minutes in group A was 8.15±0.81 and group B was 7.79±1.05 (p-value 0.02) using an unpaired t-test (Table 5).

**Table 5:** Mean Apgar at five minutes of both study groups

	Group A (n=75)	Group B (n=75)
Mean APGAR at 5 minute	8.15	7.79

No statistical correlation was seen in the distribution of NICU stay of two groups (p-value 0.53), 17.3% in group A and 21.3% in group B. Early initiation of breastfeeding by the birth companion in group A (93.3%) was higher than those in group B (73.3%) using a Chi-square test (p-value<0.01) (Table 6).

**Table 6:** Distribution of early initiation of breastfeeding

Early initiation of Breastfeeding	Group A (n=75)		Group B (n=75)		P-value
	No.	%	No.	%	
Delayed (>1 hour)	5	6.7	20	26.7	<0.01
Immediately	70	93.3	55	73.3	

**Figure 3:**
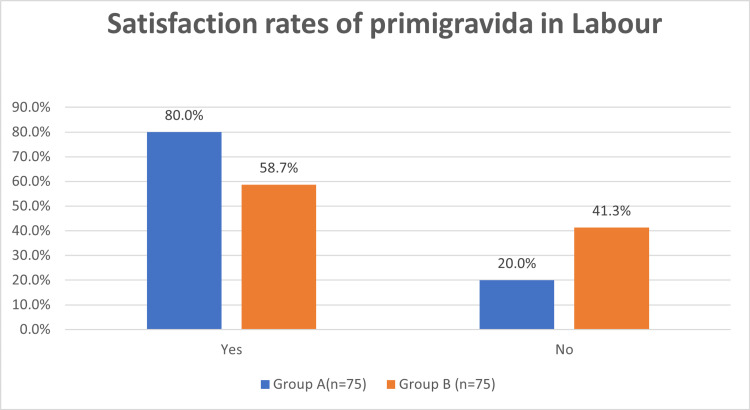
Graphical representation of the satisfaction rates of primigravida in labour

The satisfaction rate of primigravida assisted by a birth companion in labour is 80% as compared to those not assisted by a birth companion in labour receiving standard routine care is 58.7% (p-value <0.01) (Table 7, Figure 3).

**Table 7:** Satisfaction rates of primigravida in labour

	Group A(n=75)	Group B (n=75)
Yes	80.0%	58.7%
No	20.0%	41.3%

Distribution of age of birth companions with a majority between 50-59 years of age (41.3%), 40-49 years of age (25.3%), and 30-39 years of age (33.3%), using the Fisher exact test (Table 8).

**Table 8:** Age-wise distribution of birth companion

	Group A (n=75)
30-39 years	33.3%
40-49 years	25.3%
50-59 years	41.3%

**Figure 4:**
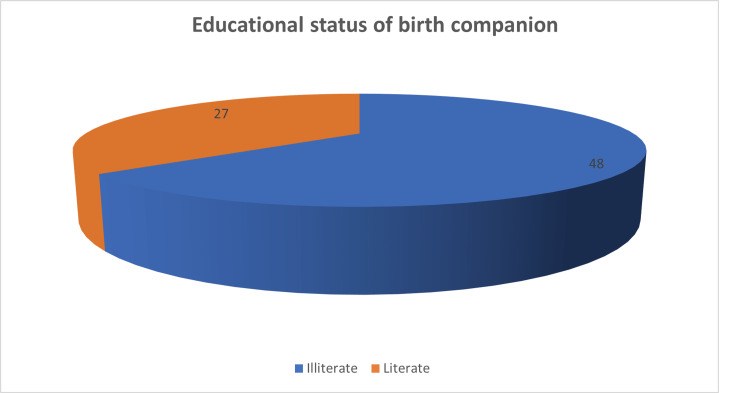
Pie chart depicting the distribution of educational status of birth companions

The mean age of birth companion who assisted primigravida in group A was 44.67±8.31 years. 64% of birth companions were illiterate and 36% were literate, using Fisher’s exact test (Table 9, Figure 4).

**Table 9:** Distribution of educational status of birth companion

	Educational status of birth companion
Illiterate	48
Literate	27
5^th^ class	24.0%
8^th^ class	12.0%

**Figure 5:**
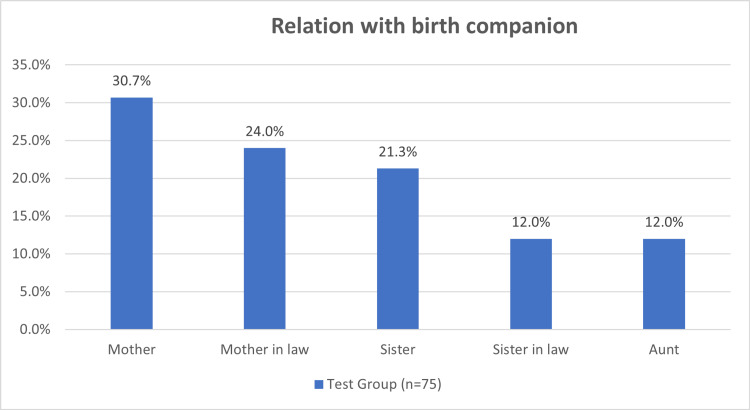
Relation of birth companion with pregnant female

The labour companionship of choice by the primigravida women were mothers-in-law (30.7%), mothers (24%), sisters-in-law (21.3%), sisters (9%) and aunts (12%) (Figure 5).

## Discussion

For all women throughout labour and delivery, the WHO [13] and the Government of India (GOI) [14] advocate birth companions. As per the updated guidelines for the COVID-19 pandemic by the GOI, birth companions can be allowed in green zones - areas with zero COVID-19 cases [15].

In our study, the sample size was 150 primigravida with term gestation, comparable with Mahshid et al. [16] with a risk ratio of 1.35, and Yuenyong et al. [17] which had a sample size of 120 to reduce the margin of error. The mean duration of the first stage of labour in group A was 9.96±3.40 hours compared to 11.95±3.11 hours in group B (p-value = 0.23). This was comparable with studies of Bohren et al. [18] and Yuenyong et al. (r = 0.48, p < 0.01) [17].

No statistical significance was seen in our study in the mean duration of 1st stage of labour in the primigravida women assisted by the birth companion (p-value = 0.23). The mean duration of 1st stage of labour in group A, which was supported by a birth companion in labour was 9.96±3.40 hours compared to 11.95±3.11 hours in group B who were not supported by a birth companion and received routine standard care in the labour room. Our findings agreed with many other studies like Hodnett et al. [11]; they concluded their study by searching Cochrane Pregnancy and Childbirth database which included 22 trials involving 15,288 women meeting inclusion criteria, showed a mean difference of shorter duration of labours by 0.58 hours in women who received one to one continuous labour support (95% CI -0.85 to -0.31). In another study conducted by Bohren et al. [9] using details from 13 trials, involving 5429 women provided evidence (low certainty evidence) that companionship during labour and childbirth probably reduces the length of labour with a mean difference of 0.69 hours shorter, 95% CI 0.34-1-04 hours shorter.

On the contrary, Scott et al. [19] in their study stated that women who received continuous social support had mean labour that was 1 hour 38 minutes shorter than the labours of women who received no additional support. However, even with a reduction of approximately 1.5 hours the mean length of labour among controls within the continuous group of studies (11.2 hours) was still longer than the value for the length of labour among the controls (6.8 hours) within the intermittent group of studies. The weighted mean differences for the length of labour are statistically significant between the two groups of studies (z = 7.9, p < 0.001). This difference could have been due to the inclusion of both primigravidas and multigravidas in the author's study as compared to only primigravidas in our study. Yuenyong et al. [17] performed a randomized control trial on 120 primiparous women and found that those women in the experimental group (with a family relative as a birth companion) had a significantly shorter duration of active labour and duration of ruptured membranes, r = 0.48, p < 0.01. Active labour for women in the experimental group was 32.2 minutes shorter (M = 268.9 minutes, SD = 88.3 minutes) than for those in the control group (M = 301.1 minutes, SD = 97.5 minutes) which supported the findings in our study.

However, some other studies like one done by Khresheh et al. [20] showed a lack of effect of support from a female relative on the duration of labour, with a mean duration of labour of 8±4.2 hours in the support group and 10±4.8 hours in the control group with a p-value of 0.180 being of no statistical significance. This result was not in keeping with the findings in our study and likely was due to the study design and the sample size.

No significant association was seen in the mean duration of the second stage of labour (p-value = 0.98) as well as in the third stage of labour (p-value = 0.72) in the presence of a birth companion compared to controls in the present study which were comparable to study conducted by Khresheh et al. [20] where there was no effect of support on duration of labour from a female relative (p-value = 0.18).

A significant association was seen in the need for augmentation with oxytocin in primigravida women assisted by labour companion as compared with controls (p-value = 0.01) which were consistent with the previous studies conducted by Scott et al. [19], which showed a significant reduction of 71% in the requirement of augmentation with oxytocin in two groups (support 55% vs controls 25%), having p-value <0.05 with CI 1.06. On the contrary, Hodnett et al. [11] in their study found no clinical significance in the need for oxytocin use in both groups (p-value = 0.93). Bruggemann [21] in the study provided evidence of no statistical significance in the need for augmentation by oxytocin (p-value = 0.65) which is contradictory to our study.

In our study, statistical significance was found in the mode of delivery in primigravida women assisted by a birth companion with the majority of women (89.3%) having normal delivery outcomes, compared to the group not assisted by a birth companion (76%). There is a reduction in caesarean section rates by 10.6% in the groups assisted by birth companions (10.7%) versus those who did not receive labour support (21.3%). Primigravida women who received continuous labour support from a family relative had a more spontaneous normal vaginal delivery, lesser caesarean section rate, and lesser instrumental deliveries (0% versus 2%), with a statistically significant association (p-value = <0.05).

All these findings were supported by several studies by Scott et al. [19] in a study performed on 11 trials that found a 51% reduction in caesarean births (p <0.05) and a 57% reduction in forceps birth (p<0.05). Pascali-Bonaro et al. [22] conducted a study that found 26% reduced caesarean rates and 41% less likely to give birth with vacuum extraction or forceps in women supported by a companion in labour. Hodnett et al. [11] compared continuous support during labour with usual care and found that they were less likely to have a caesarean birth (RR 0.90, 95% CI 0.85 to 0.96). Bohren et al. [9] included a total of 24 trials involving 15,347 women and found allocated to continuous labour were more likely to have a spontaneous vaginal birth and less likely to have a caesarean birth (average RR 0.75, 95% CI 0.64 to 0.88). In a study done by Kabakian-Khasholian et al. [23] on two groups, (p-value = 0.98) with one group showing the study found that drop in caesarean section births from 22% to 11% after implementing labour companionship (p = 0.001; 95% CI: 0.09-0.13).

The secondary outcome of our study has been evaluated by noting Apgar scores at five minutes of age, and NICU stays and comparing their overall satisfaction rates with childbirth experience by asking questions to each woman through a pre-designed questionnaire after 12 hours of delivery. We found statistically significant overall satisfaction rates among women supported by a birth companion during labour and delivery, especially in a government hospital like Ambedkar Hospital with the increased patient-to-bed ratio making it very difficult for the health care personnel to provide adequate care to each woman in labour.

In the present study, a significant association was found in our study in mean Apgar at five minutes in primigravida females supported by a birth companion during her childbirth with the value of 8.15±0.81 as compared to women who received routine care with a value of 7.79±1.05 (p<0.05), which was comparable in studies by Hodnett et al. [11], which showed that females having continuous support during labour were less likely to have a baby with a low five-minute APGAR score (RR 0.69, 95% CI 0.50 to 0.95). However, no significant association was seen in the study done by Bruggemann et al. [21] on the Apgar score at five minutes (p=0.68), which does not correlate with our study. In our study, there was no association seen in the admission of the babies to NICU in both the groups with p-value = 0.53, being statistically insignificant.

A significant association was seen between the presence of a birth companion and initiation of breastfeeding with p-value <0.01, having early initiation of breastfeeding immediately after birth in 93.3% of females by a birth companion, as compared to females who were not supported by a birth companion with only 73.3% had early initiation of breastfeeding. These findings are contradictory to the study by Hodnett et al. [11] which showed no apparent impact on breastfeeding by continuous support. 

In our study, as per results obtained through a pre-designed questionnaire asked individually to women in both groups after 12 hours of delivery, we found statistically significant satisfaction rates with their childbirth experience of 80% in primigravida women assisted by labour companion as compared to just 58.7% in women receiving routine standard care in labour room without a birth companion (p <0.01). The findings of our study were strongly supported by the study of Bruggemann et al. [21] with women in the support group being more satisfied with labour (median 88.0 vs 76.0, p <0.0001) and delivery (median 91.4 versus 77.1, p <0.0001).

Pascali-Bonaro et al. [22] in their review which included 15 studies found that the women who received continuous support were 36% less likely than women who did not receive continuous support to report dissatisfaction or a negative rating of their birth experience. Hodnett et al. [11] in their study compared continuous support during labour with usual care and found women allocated to labour support were less likely to report dissatisfaction (RR 0.69, 95% CI 0.59 to 0.79). Kabakian-Khasholian et al. [23] found that women’s satisfaction during childbirth improved from 113.39 to 115.98 points (p=0.000; 95% CI:5-8). Yuenyong et al. [17] showed a significant maternal satisfaction rate with her childbirth experience when supported by a close female relative with a mean (SD) value of 53.6 (8.4) in the support group compared to controls 47.9 (10.7) on the LAS scale (p-value <0.001).

In our study, the majority of primigravida women delivered by spontaneous normal vaginal delivery with shorter labour duration and lower need for oxytocin augmentation, having lower caesarean rates in the presence of a birth companion during labour and childbirth when compared to women receiving standard care. These mothers had higher Apgar scores at five minutes and started breastfeeding earlier. This study also found that primigravida women who were supported by a birth partner had higher overall satisfaction rates (Table 10).

**Table 10:** Comparison of a few studies with this study’s outcomes

Authors	Spontaneous Normal vaginal birth in birth companion	Caesarean Birth in presence of birth companion	Mean Duration of labour in presence of birth companion	Satisfaction rates
Kabakian et al. (2013) [23]	89% vs 78%	11% vs 22%	Decreased significantly by 30 minutes p=0.001	115.98 points to 113.39 points
Yuenyong et al. (2012) [17]	56.9% vs 55.4%	10% vs 25%	Active labour 32.2 minutes shorter (268.9 minutes vs 301.1 minutes)	53.6 points vs 47.9 points (p<0.01 on LAS scores)
Hodnett et al. (2011) [11]	RR 1.08, 95% CI 1.04 to 1.12	RR 0.78, 95% CI 0.67 to 0.91	Mean deviation 0.58 hours shorter	Less likely to report dissatisfaction (RR 0.69, 95% CI 0.59 to 0.79)
Bruggemann et al. (2007) [21]	94 vs 95	11 vs 12 (RR 0.93, 95% CI 0.43-2.02)	Mean deviation 0.40 hours shorter	Median 88.0 versus 76.0 (p-value <0.0001)
This study	89.3% vs 76% P value 0.03 (significant)	10.7% vs 21.3%	Mean duration shorter by 1 hour 30 minutes	80% vs 58.7% P-value <0.01

The age of birth companions (n=75) who supported pregnant women in group A in our study ranged between 30 and 59 years of age with the mean age of birth companion was 44.67±8.31 years. The majority (41.3%) of labour companions aged between 50-59 years of age, with 33.3% in the age group 30-39 years of age and 25.3% in 40-49 years of age. More than half of the family relatives who provided support to women in group A were illiterate women (64%). The persons who were most often chosen as the close female relative to provide companionship during labour and delivery were mothers-in-law (30.7%), mothers (24%), and sisters-in-law (21.3%). Their support was provided to the woman continuously and they left the woman’s side only sporadically.

This study was done from 2019 to 2021. With the declaration of the COVID-19 pandemic on March 12, 2020, the option of permitting birth companions in the labour ward was not implemented due to the practical risk of spreading the disease and the inability to maintain social distance due to space restrictions. The study was withheld during the peak of COVID-19 cases when vaccines were unavailable. No significant results were seen on the mean duration of labour due to the small sample size. There were times when proper privacy could not be maintained due to space constraints and overcrowded wards.

## Conclusions

Birth companions are women who have experienced the process of labour and provide continuous one-to-one support to other women experiencing labour and childbirth. Clinically significant benefits of the assistance have been demonstrated in research, including shorter labour time, greater rates of spontaneous vaginal birth, decreased caesarean section and intrapartum analgesia, and increased satisfaction with childbirth experiences. Labour companionship should be routinely advised to all pregnant women throughout labour and delivery to provide RMC, particularly at government hospitals with high patient-to-bed ratios to promote a positive childbirth experience. Tertiary centres in India face issues such as space limitations, inadequate privacy, a high patient load, limited staff, and insufficient rules, and guidelines, which should be addressed through training and programmes organized through the Laqshya initiative and help in better implementation of birth companionship.
